# A Two-Step Process Gets mRNA Loaded and Ready to Go

**DOI:** 10.1371/journal.pbio.1001047

**Published:** 2011-04-19

**Authors:** Richard Robinson

**Affiliations:** Freelance Science Writer, Sherborn, Massachusetts, United States of America

**Figure pbio-1001047-g001:**
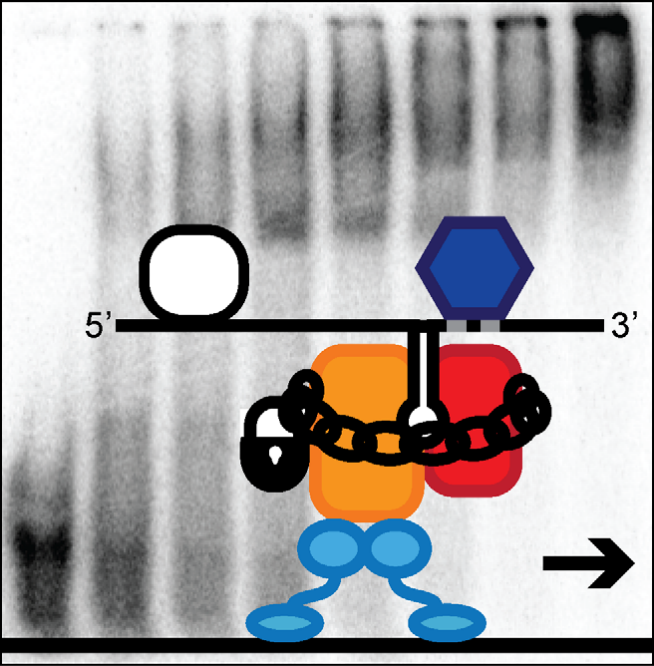
***In vitro*** **reconstitution experiments with an mRNA-transport complex from yeast identify a quality control step for the incorporation of correct transcripts into mature transport particles.**

[Fig pbio-1001047-g001]Proteins are the workhorses of the cell, but to get the most work out of them, they need to be in the right place. In neurons, for example, proteins needed at axons differ from those needed at dendrites, while in budding yeast cells, the daughter cell needs proteins the mother cell does not. In each case, one strategy for making sure a protein gets where it belongs is to shuttle its messenger RNA to the right spot before translating it.

The destination for such an mRNA is encoded in a set of so-called “zipcode” elements, which loop out of the RNA string to link up with RNA-binding proteins. In yeast, these proteins join up with a myosin motor that taxis the complex to the encoded location. While this general picture has become clear in the past several years, details of the transport complex itself have remained murky. In this issue of *PLoS Biology*, Marisa Müller, Dierk Niessing, and colleagues show that the complex forms in stages, with the final protein added only in the cytoplasm, an event crucial to ensuring the transport complex doesn't pick up passengers who weren't meant to travel.

The authors began by showing that a known zipcode element binding protein, called She2p, bound in a similar way to RNAs without the elements as to those with them, suggesting that the job of keeping non-transported RNAs out of the transport complex fell to another protein. Tests of another known complex protein, called Puf6p, were negative, leading the authors to look elsewhere.

One candidate was She3p, known to link the complex to the myosin motor. They found that She3p co-purified with She2p, indicating they linked together, and that together they bound tightly to zipcode-containing mRNAs, but not to other RNAs without the address sequences. She3p also interacted with RNAs by itself, albeit weakly, which was a surprise, since its sequence bore no resemblance to other RNA-binding proteins. When She2p was mutated to prevent its linkage to RNA, the ability of the RNA–protein complex to reach its destination was impaired.

She2p binds to its target RNAs in the nucleus, but She3p was thought to be a cytoplasmic protein. To test that belief, they examined its distribution in a cell with a temperature-sensitive defect in nuclear export, and saw that even at high temperatures, when nuclear export was shut down, She3p was found only in the cytoplasm, indicating that it, unlike She2p, waits for the nascent transport complex to enter the cytoplasm before it binds on.

Based on their results, the authors propose a two-step model of transport complex formation. Within the nucleus, She2p binds to the mRNA as it is transcribed, and then shuttles it to the cytoplasm. She2p binds loosely and promiscuously, though, catching up mRNAs both with and without zipcodes. Once in the cytoplasm, She3p joins on, tightening the grip on mRNAs that contain zipcodes while booting out those without them. With the myosin motor attached to She3p, the complex motors off to its destination elsewhere in the cell.

The results in this study indicate that quality control in mRNA transport relies on a reciprocal action: the complex proteins together ensure that only those mRNAs with a destination tag are incorporated into the transport complex, and the mRNA, by binding to each of the proteins in the complex, ensures that all are on board before the journey starts.

These findings are an important step forward to understand how mRNAs are transported to their chosen destination in yeast, and opens up the possibility that a similar strategy is followed in other organisms.

**Müller M, Gerhard Heym R, Mayer A, Kramer K, Schmid M, et al. (2011) A Cytoplasmic Complex Mediates Specific mRNA Recognition and Localization in Yeast. doi:10.1371/journal.pbio.1000611**

